# Supplementary material for: Wafer-scale integration of two-dimensional perovskite oxides towards motion recognition
Source: Nat Commun. 2024 Oct 10;15:8789. doi: 10.1038/s41467-024-52840-2 (PMC11467426; doi:10.1038/s41467-024-52840-2)
Supplement: Supplementary file 1 — Supplementary Information [file 41467_2024_52840_MOESM1_ESM.pdf]

Supplementary Information

Wafer-scale integration of two-dimensional perovskite oxides towards motion recognition

Ming Deng[1], Ziqing Li[2]*, Shiyuan Liu[3], Xiaosheng Fang[1,2]* & Limin Wu[1,4]*

[1]Department of Materials Science and State Key Laboratory of Molecular Engineering of Polymers, Fudan University, Shanghai 200438, P. R. China

[2]Shanghai Frontiers Science Research Base of Intelligent Optoelectronics and Perception, Institute of Optoelectronics, Fudan University, Shanghai 200433, P. R. China.

[3]Optical Fiber Research Center, Department of Materials Science, Fudan University, Shanghai 200433, P. R. China

[4]College of Chemistry and Chemical Engineering, Inner Mongolia University, Hohhot 010021, P. R. China

*Corresponding author.

e-mail: lzq@fudan.edu.cn; xshfang@fudan.edu.cn; lmw@fudan.edu.cn.

Supplementary Information

This supplementary information includes:

Supplementary Figure 1 to 27

Supplementary Table 1 and 2

Supplementary Note I to V

Supplementary References

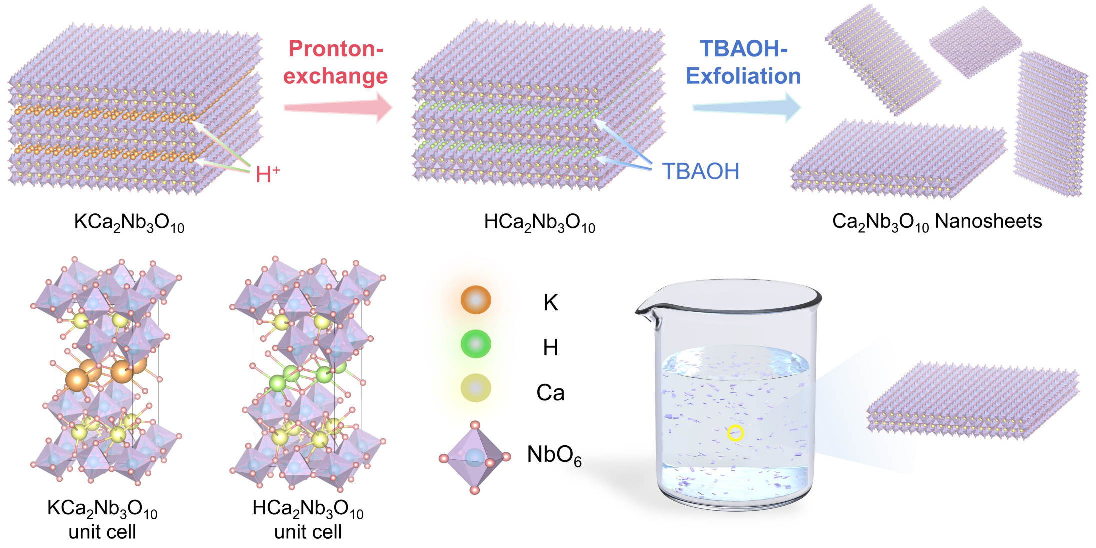

Supplementary Fig. 1 | The synthesis process of $Ca_2Nb_3O_{10}$. Top-down synthesis strategy for the delamination of calcined DJ-phase oxide perovskite crystals through mechanical cleavage and ion-exchange exfoliation and unit cell structures of $KCa_2Nb_3O_{10}$ and $HCa_2Nb_3O_{10}$. Note that after acid converts $KCa_2Nb_3O_{10}$ to $HCa_2Nb_3O_{10}$, the protons in $HCa_2Nb_3O_{10}$ are then replaced by TBAOH treatment to achieve exfoliation, and finally TBA^+ cations are washed away to produce $Ca_2Nb_3O_{10}^-$ nanosheets. In the above process, negative charges are introduced into the nanosheets due to the stripping of interlayer cations, which is proved in Supplementary Fig. 2.

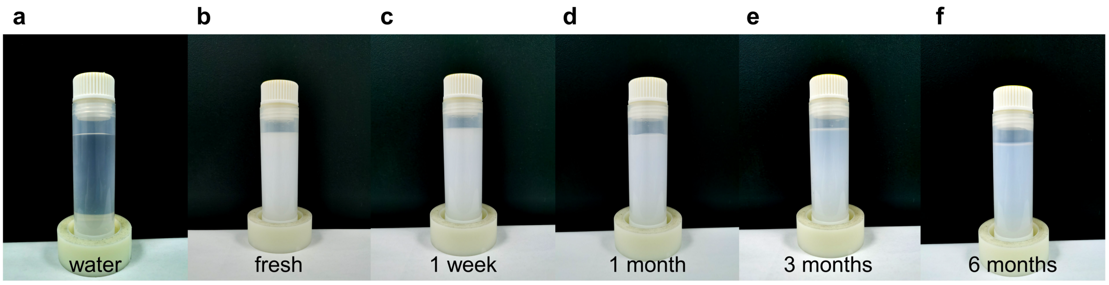

Supplementary Fig. 2 | Photographs of CNO nanosheets in aqueous solution. (**a**) control pure water, (**b**) fresh CNO nanosheets solution, (**c**) after placement for 1 week, (**d**) after placement for 1 month, (**e**) after placement for 3 months, (**f**) after placement for 6 months. After several months of placement, the nanosheets remains dispersed in solution without obvious settling or aggregation, proving the existence of repulsive forces between the nanosheets.

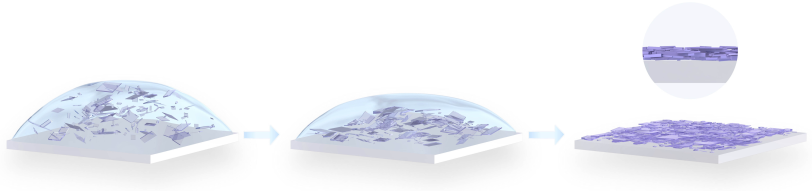

Supplementary Fig. 3 | Schematic illustration of the film-formation process of D-CNO film in traditional top-down strategy.

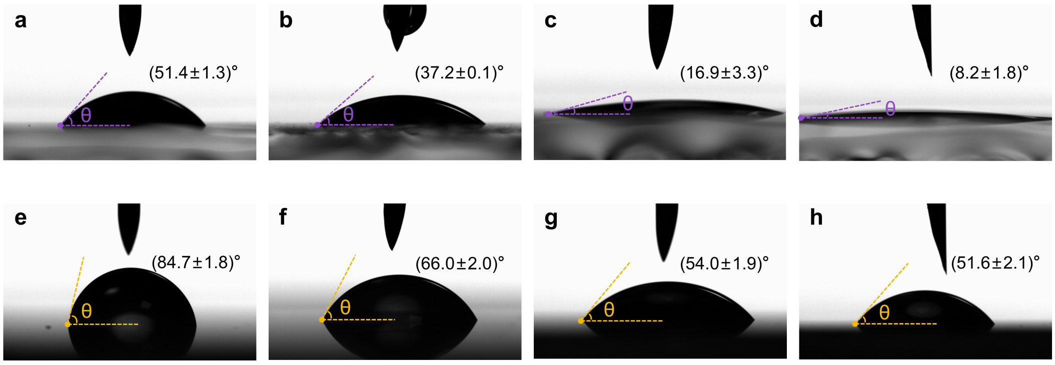

Supplementary Fig. 4 | Contact angle measurement. Contact angle images of solvent mixture droplets (CNO precursor solution, deionized water and anhydrous ethanol) on glass (**a**-**d**) and Parylene-C (**e**-**h**) with volume ratio of water: ethanol=2:0 (**a** and **e**), water: ethanol=2:1 (**b** and **f**), water: ethanol=2:2 (**c** and **g**), water: ethanol=2:3 (**d** and **h**). As the concentration of ethanol increases, the contact angles of the solvent mixture droplets on both glass and Parylene-C decrease, suggesting that the increase in ethanol reduces the surface energy of the solvent and improves its wettability with the substrates.

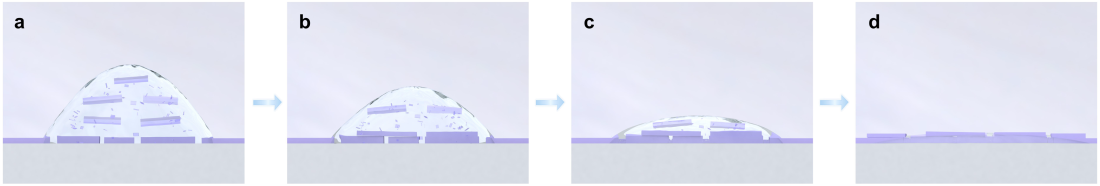

Supplementary Fig. 5 | Complement to the COAF process: void-filling process between large nanosheets. (**a**) Nanosheets with different sizes dispersed in the micro-droplet. (**b**) Larger nanosheets settle as the solution evaporates. (**c**) Tinier nanosheets enter the blank space formed between larger nanosheets as the solvent evaporates and percolates. (**d**) Tinier nanosheets fill in the blank spaces and voids to form continuous and uniform film.

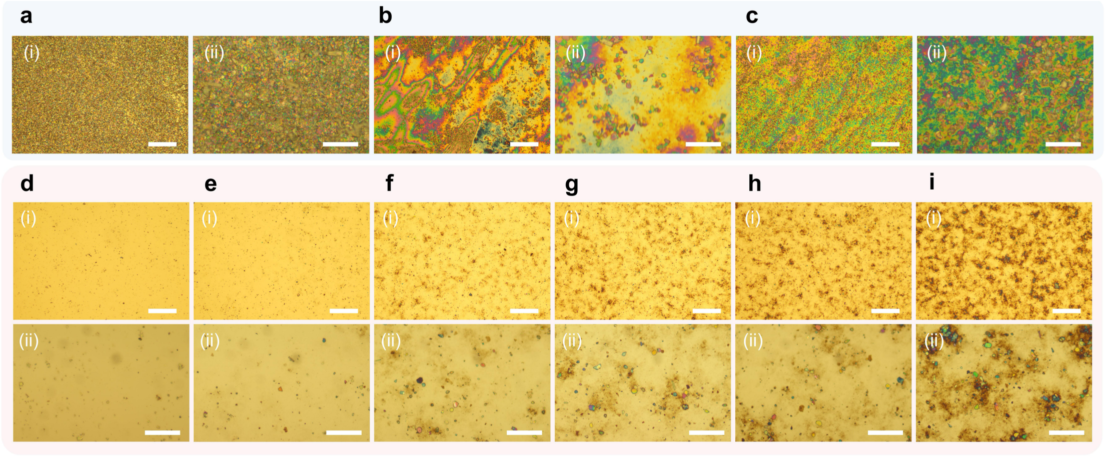

Supplementary Fig. 6 | Optical photographs of CNO films. a-i Optical photographs of D-CNO film at center position on p-type Si wafer (**a**), D-CNO film at edge position (**b**), Spin-coated CNO film (**c**), S-CNO 4 mL film (**d**), S-CNO 6 mL film (**e**), S-CNO 8 mL film (**f**), S-CNO 10 mL film (**g**), S-CNO 12 mL film (**h**) and S-CNO 14 mL film (**i**). Scale bar: (i) 100 μm, (ii) 25 μm. Note that it can be seen that there is much and obvious residual solvent in the dropped-coated (a and b) and spin-coated films (c). In contrast, very little residual solvent can be observed in our spray-coated films (d-i), which may greatly eliminate the negative impact of solvent on the device performance. The significantly fewer residual solvent to the rapid volatilization of the mixed solvent, which is caused by the addition of low-boiling anhydrous ethanol and the large-area nature of the spray-coating process.

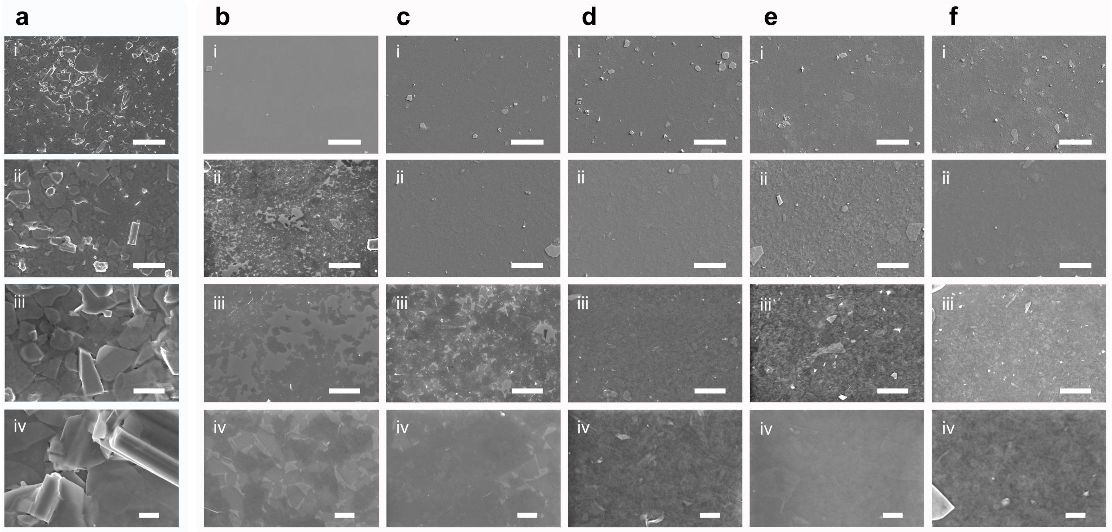

Supplementary Fig. 7 | SEM images of CNO films. a-f, SEM images of D-CNO film (**a**), S-CNO 4 mL film (**b**), S-CNO 6 mL film (**c**), S-CNO 8 mL film (**d**), S-CNO 10 mL film (**e**) and S-CNO 12 mL film (**f**). Scale bar: (i) 10 µm, (ii) 4 µm, (iii) 2 µm, (iv) 500 nm. Note that large size nanosheets are observed on the surfaces of both D-CNO and S-CNO films, which are formed by incomplete exfoliation. And the nanosheets on the surface of D-CNO seem to be more and larger, which we believe is due to: (1) Difference in the film-formation process. Large nanosheets in S-CNO films tend to be buried inside and form uniform surfaces, while that in D-CNO films are exposed on the surfaces; (2) As relatively more precursor solution per unit area is needed to prepare the D-CNO film, there will be more large nanosheets in them than in S-CNO films, while the large nanosheets on the S-CNO films appear to be more dispersed.

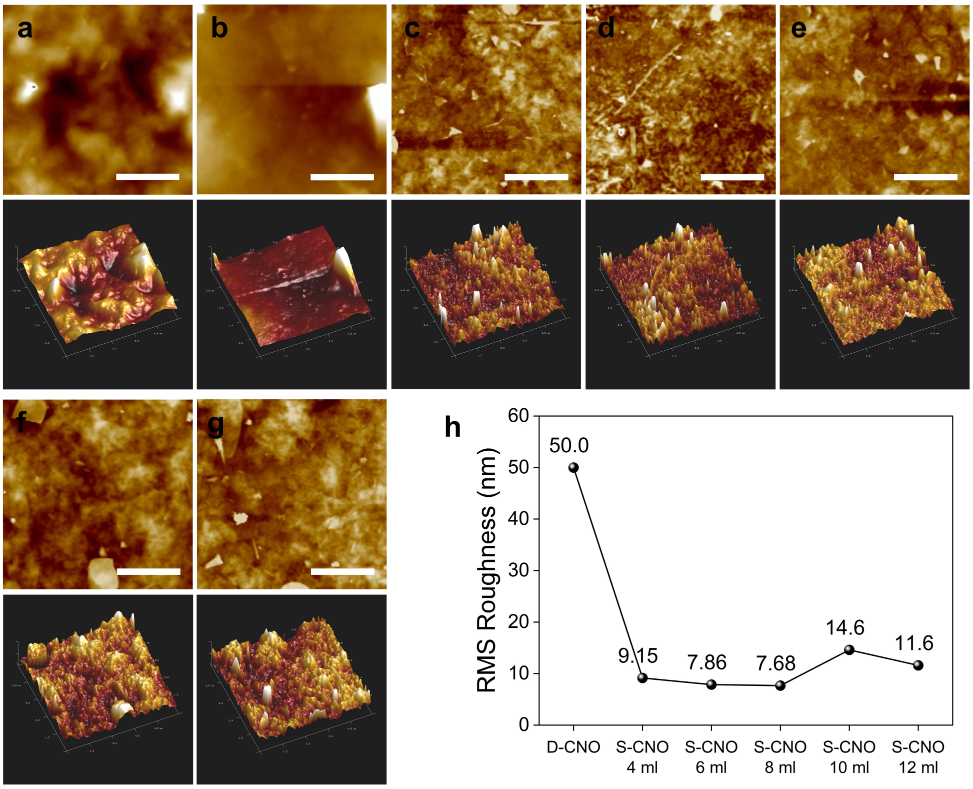

Supplementary Fig. 8 | Surface roughness characterization of CNO films. 2D AFM images with scale bars of 2μm and the corresponding 3D AFM images of (**a**) D-CNO film at center position, (**b**) D-CNO film at edge position, (**c**) S-CNO 4 mL film, (**d**) S-CNO 6 mL film, (**e**) S-CNO 8 mL film, (**f**) S-CNO 10 mL film and (**g**) S-CNO 12 mL film. (**h**) RMS roughness characterization of the fabricated CNO films.

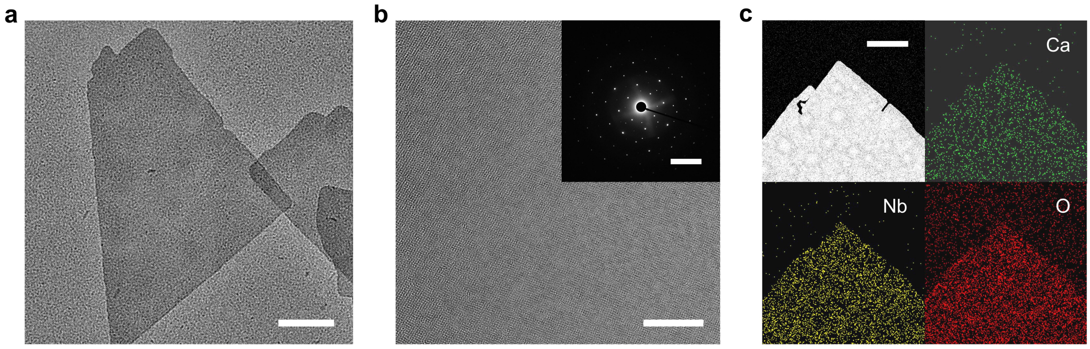

Supplementary Fig. 9 | TEM characterization of CNO nanosheet. a, TEM image of a single CNO nanosheet. Scale bar: 100 nm. **b**, HRTEM image of the CNO nanosheet. Scale bar: 10 nm. Inset shows the corresponding SAED pattern. Scale bar: 5 1/nm. **c**, TEM image and corresponding EDS mapping of the Ca, Nb, and O elements. Scale bar: 200 nm.

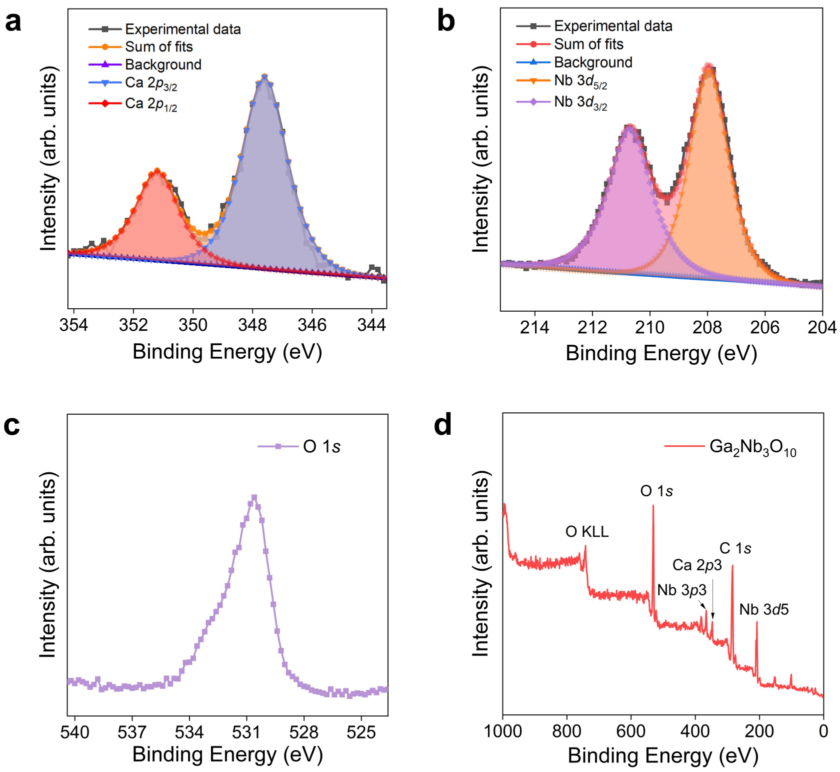

Supplementary Fig. 10 | XPS characterization of S-CNO films. a-c, XPS spectra of (**a**) Ca 2*p*, (**b**) Nb 3*d* and (**c**) O 1*s*. **d**, XPS survey spectrum of CNO film.

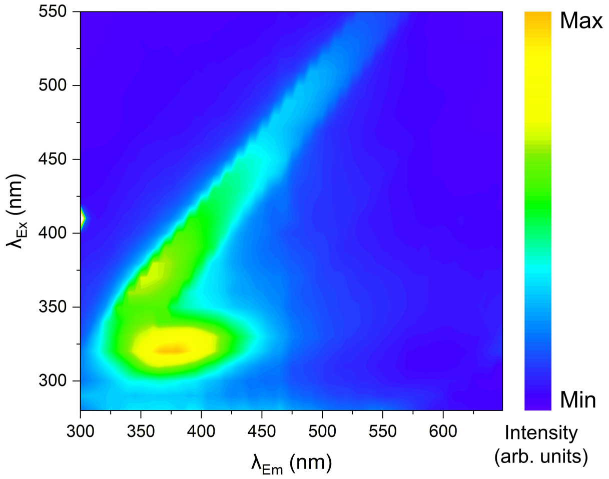

Supplementary Fig. 11 | The excitation-emission matrix spectrum of the S-CNO 10 mL film.

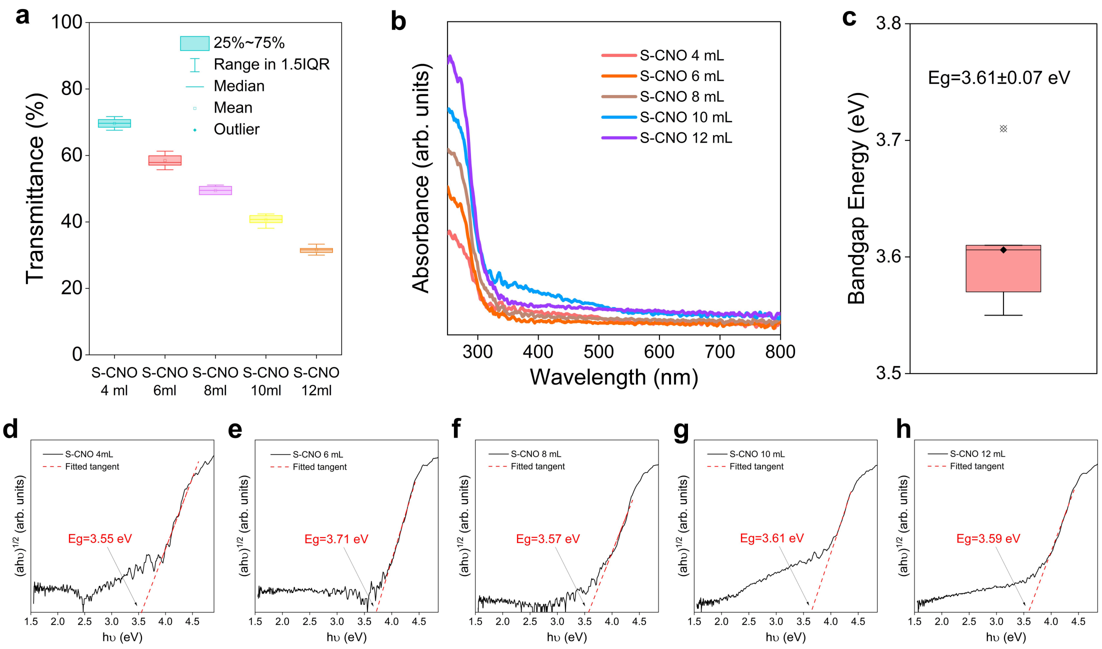

Supplementary Fig. 12 | Absorbance and transmittance characterization of S-CNO films. **a**, The transmittance of S-CNO films with different thicknesses tested under 280 nm UV. **b**, UV-vis absorption spectrum of S-CNO films with different thicknesses. **c**, Statistics of bandgap for S-CNO films with different thickness. The boxplot is an acknowledged statistical approach, showing the maximum value, upper quartile, median, lower quartile and the minimum value. **d**-**h**, Tauc plot of S-CNO films with different thickness. Note that the error bars represent the standard deviation and are calculated based on the variation in the data of transmittance and bandgap energy.

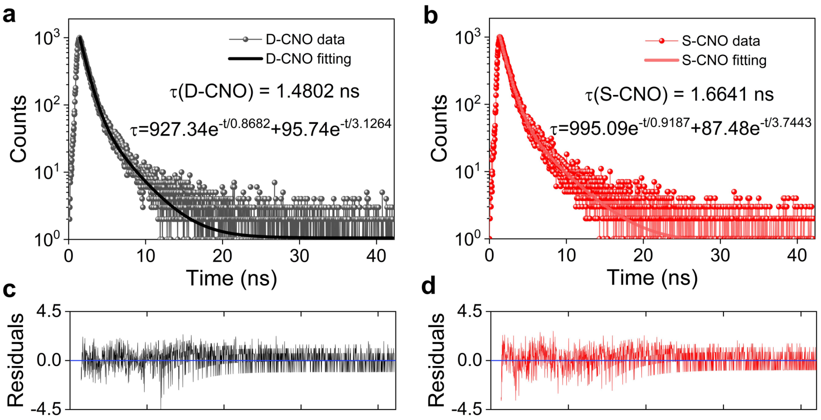

The fitted parameters of the TRPL spectrum.

		Parameter 1	Parameter 2	Average
S-CNO	A [Std. Dev]	995.09 [8.673]	87.48 [5.084]	-
	τ (ns) [Std. Dev]	0.9187 [0.012]	3.7443 [0.099]	1.6641
D-CNO	A [Std. Dev]	927.34 [9.212]	95.74 [6.949]	-
	τ (ns) [Std. Dev]	0.8682 [0.013]	3.1264 [0.095]	1.4802

Supplementary Fig. 13 | TRPL characterization of CNO films. a, **b**, Detailed TRPL spectrum and of fitted diexponential decay curve of (**a**) D-CNO and (**b**) S-CNO films. **c**, **d**, Residual of fitted curves of (**c**) D-CNO and (**d**) S-CNO films. The table shows the fitted parameters of the TRPL spectrum. Note that the decay process consists of two components, including a fast decay process (τ_1) and a slow decay process (τ_2). While τ_1 is usually thought to be attributed to the surface recombination and the shallow-trap dominated trapping/de-trapping process, τ_2 is related to the internal recombination and the deep-trap dominated trapping/de-trapping process[1-3]. Compared with the slow decay of D-CNO film (τ_1 = 0.8682 ns, τ_2 = 3.1264 ns, τ_{ave} = 1.4802 ns), the τ_1, τ_2, and τ_{ave} of S-CNO film (τ_1 = 0.9178 ns, τ_2 = 3.7443 ns, τ_{ave} = 1.6641 ns) increased by 5.8%, 19.8%, and 12.4%, respectively. This demonstrates an overall reduction in traps, especially the significant reduction in deep trap and internal defects inside the film.

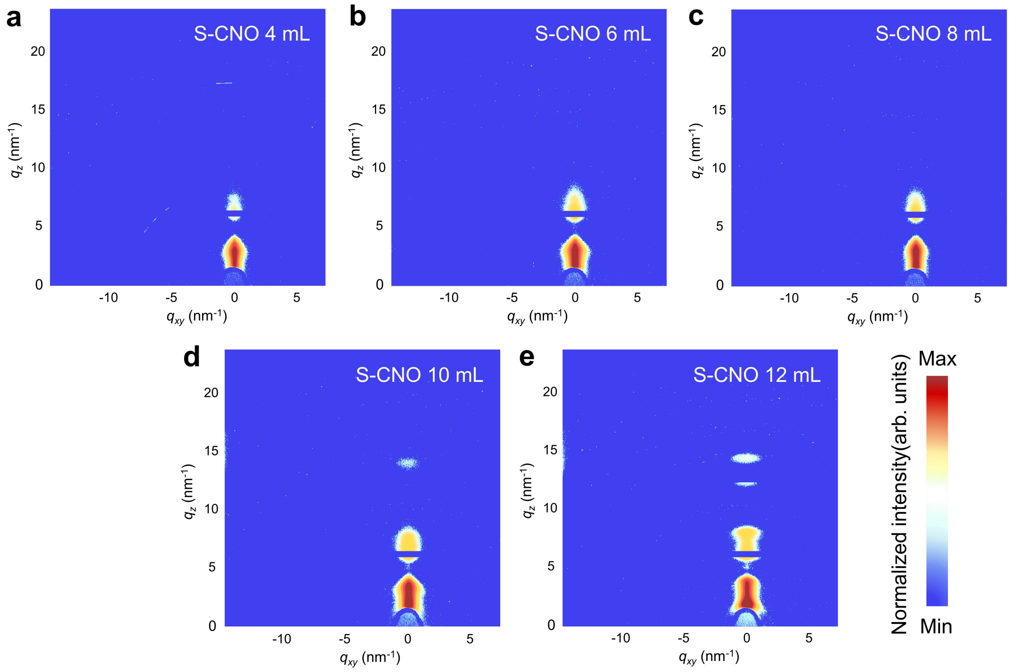

Supplementary Fig. 14 | GIWAXS patterns of S-CNO films with different thicknesses. a-e, GIWAXS patterns of (**a**) S-CNO 4 mL film, (**b**) S-CNO 6 mL film, (**c**) S-CNO 8 mL film, (**d**) S-CNO 10 mL film, and (**e**) S-CNO 12 mL film with incident angles of 0.1°.

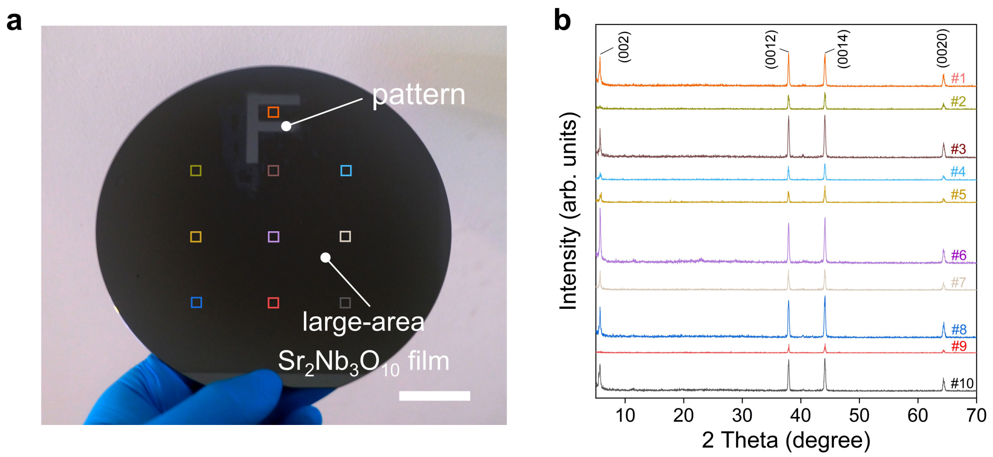

Supplementary Fig. 15 | Characterization of large-area $Sr_2Nb_3O_{10}$ film. a, Photograph of integrated large-area patterned $Sr_2Nb_3O_{10}$ film on a 6-inch n-type Si wafer. Scale bar: 3 cm. **b**, XRD patterns of $Sr_2Nb_3O_{10}$ nanosheets at different positions on the fabricated $Sr_2Nb_3O_{10}$ film.

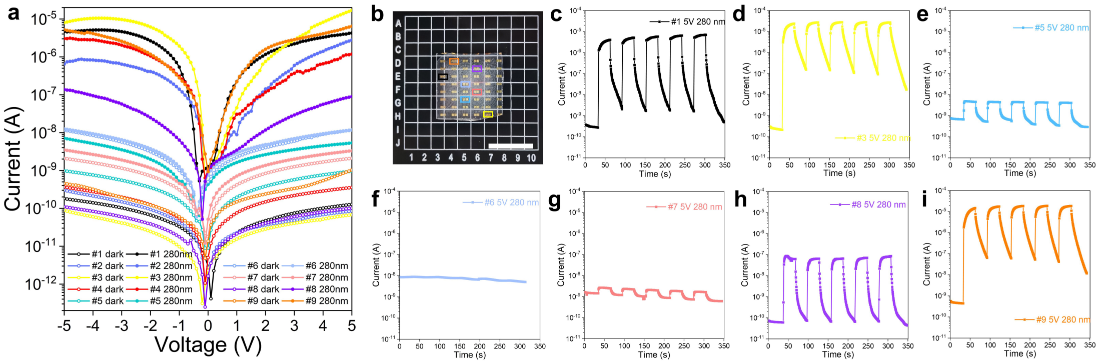

Supplementary Fig. 16 | Optoelectronic measurements of the D-CNO film. a, Semilogarithmic *I-V* curves of devices at various positions on D-CNO film. Scale bar: 1 cm. **b**, Photograph of devices on D-CNO film. **c-i**, Semilogarithmic *I-t* curves of device (**c**) #1, (**d**) #3, (**e**) #5, (**f**) #6, (**g**) #7, (**h**) #8 and (**i**) #9 under 1 V bias and 280 nm UV illumination.

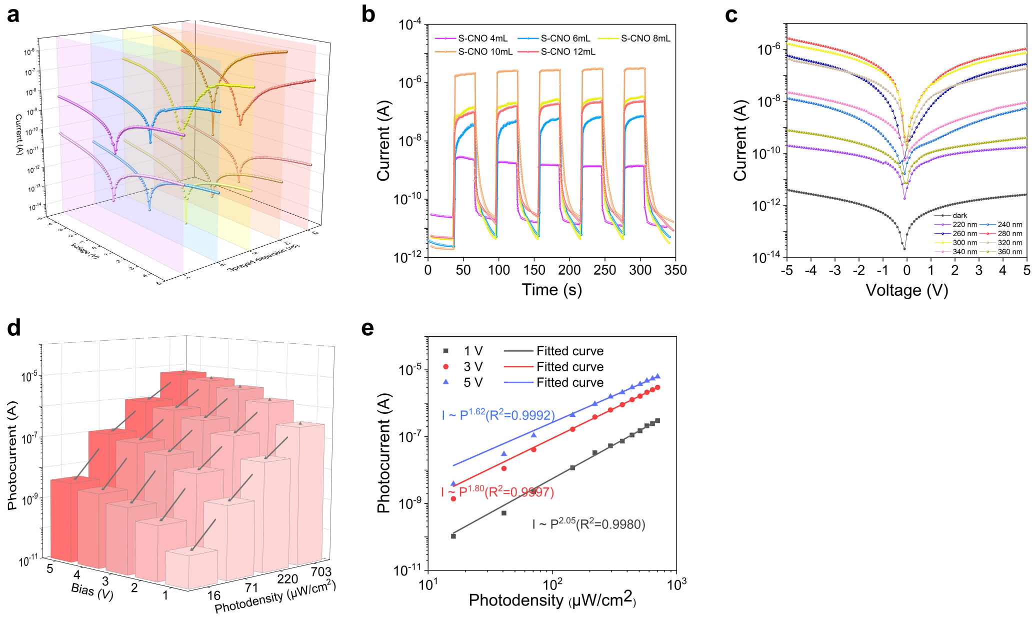

Supplementary Fig. 17 | Optoelectronic measurements of the S-CNO films. a, Semilogarithmic *I-V* curves of S-CNO film devices with different thicknesses in dark and under 280 nm UV illumination. **b**, Semilogarithmic *I-t* curves of S-CNO film devices with different thicknesses under 280 nm UV on/off switching. **c**, Semilogarithmic *I-V* curves of S-CNO 10 mL device under dark and UV illumination with different wavelengths. **d**, Photocurrent under different applied biases is plotted against photodensities under 280 nm illumination. **e**, Photocurrent as a function of incident light density and their corresponding power fits at 1 V, 3 V and 5 V bias.

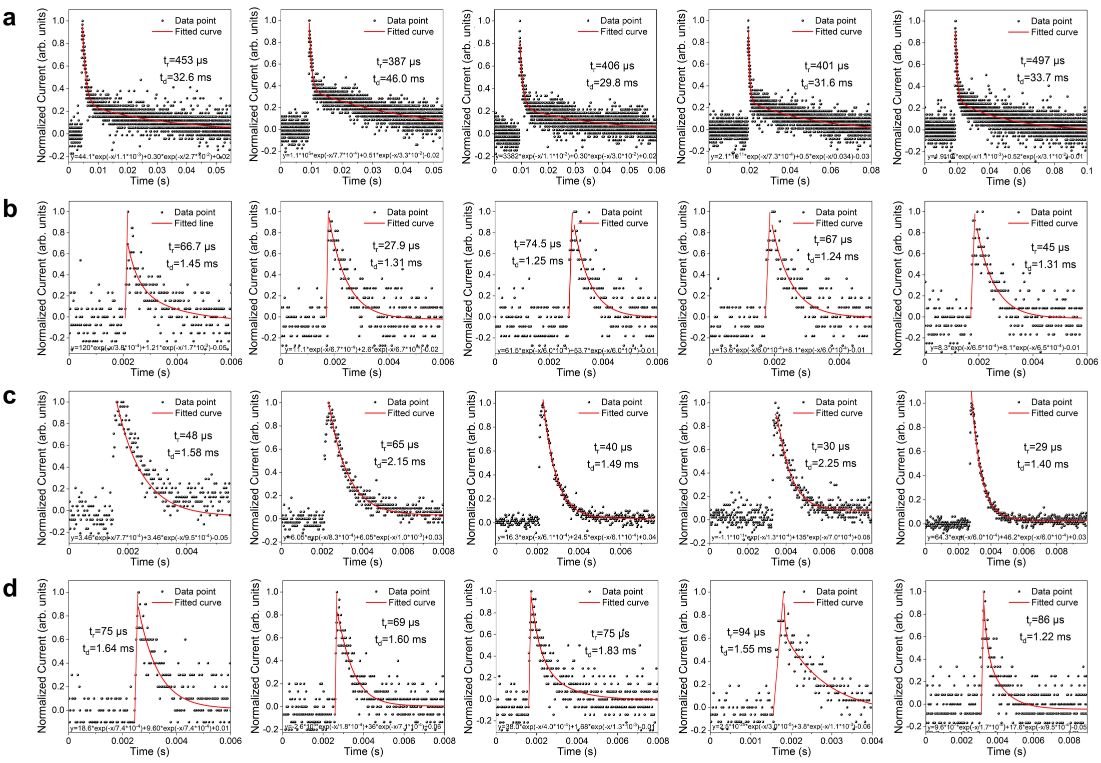

Supplementary Fig. 18 | Transient response speed measurements of CNO films. a-d, Time-resolved photoresponse of D-CNO and S-CNO devices under 10 Hz 355 nm laser pulse at 1 V bias. (**a**) D-CNO devices, (**b**) S-CNO 8 mL devices, (**c**) S-CNO 10 mL devices, (**d**) S-CNO 12 mL devices.

The t_r and t_d of D-CNO devices are (429 ± 45) μs and (34.7± 6.5) ms.

The t_r and t_d of S-CNO 8 mL devices are (56.2 ± 19.3) μs and (1.31 ± 0.08) ms.

The t_r and t_d of S-CNO 10 mL devices are (42.4 ± 14.8) μs and (1.77 ± 0.40) ms.

The t_r and t_d of S-CNO 12 mL devices are (79.8 ± 10.0) μs and (1.57 ± 0.22) ms.

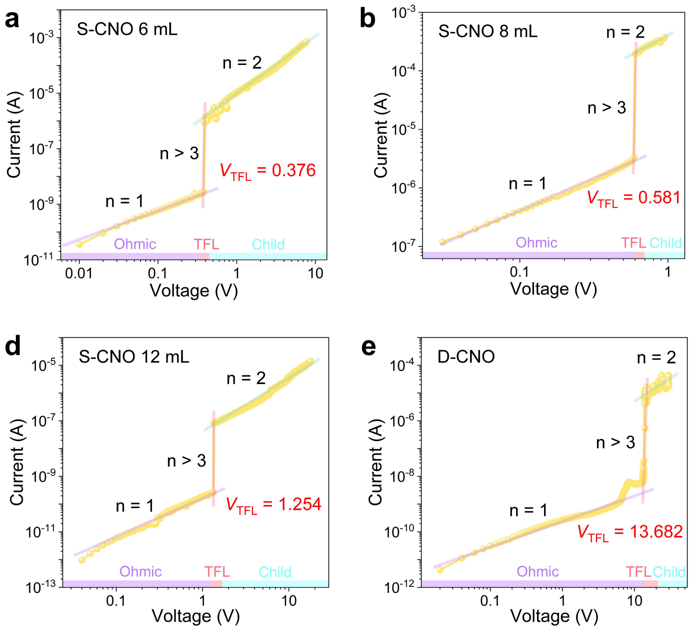

Supplementary Fig. 19 | SCLC tests of CNO films. a-e, Dark *I-V* characteristics of hole-only devices measured using the space-charge-limited current (SCLC) method. (**a**) S-CNO 6 mL device, (**b**) S-CNO 8 mL device, (**c**) S-CNO 10 mL device, (**d**) S-CNO 12 mL device, and (**e**) D-CNO device.

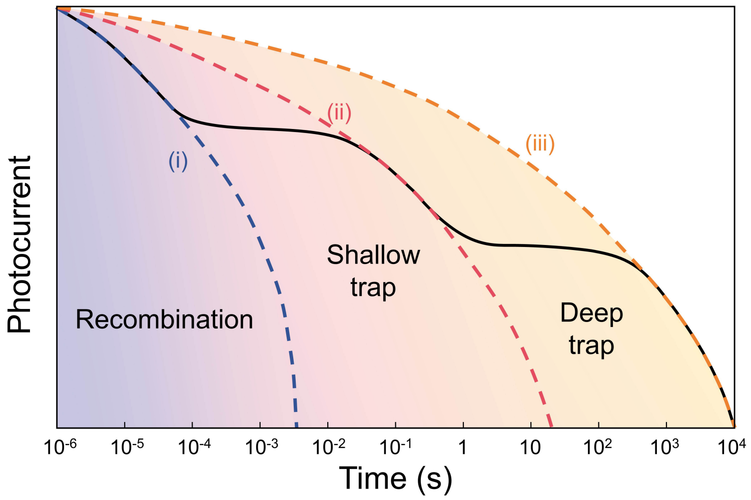

Supplementary Fig. 20| Schematic of change with time of photocurrent decay of semiconductors. The decay process can be defined into three types. Decay curve i represents a rapid decay process dominated by recombination within 10^{-3} s; curve ii represents a decay process dominated by shallow traps within $10^{-3} \sim 10$ s; curve iii represents a slow decay process dominated by deep traps within about $10 \sim 10^4$ s.

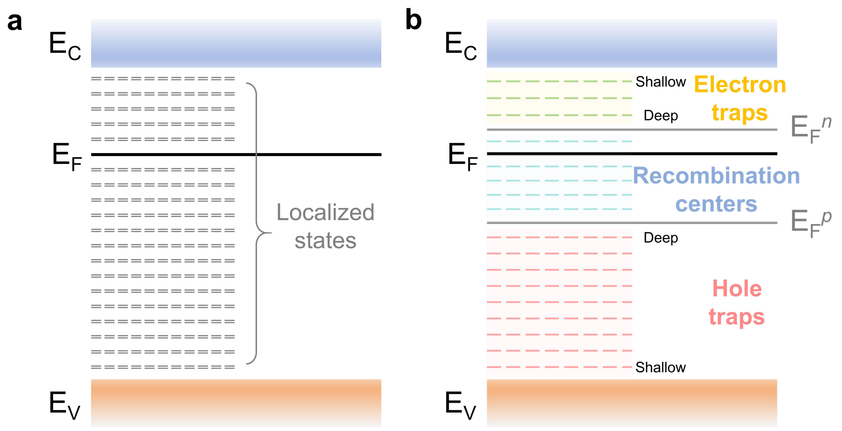

Supplementary Fig. 21 | Localized states in semiconductors. a, **b**, Schematic of localized states in semiconductors in dark (**a**), traps states and recombination centers under illumination (**b**).

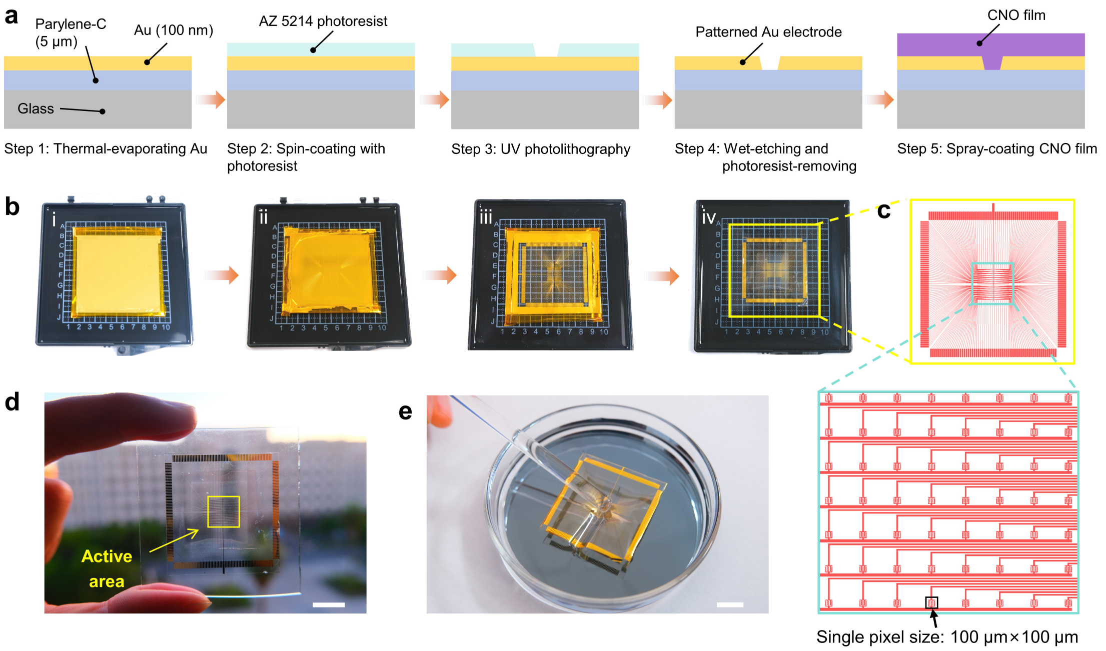

Supplementary Fig. 22 | Fabrication process of 256-pixel photodetector array. a, **b**, Schematic illustration (**a**) and photographs (**b**) of the devices array fabrication process. **c**, Schematic of active area and pixel distribution. **d**, **e**, Photographs of fabricated devices array on glass substrate (**d**) and after being peeled off (**e**) with scale bars of 1 cm.

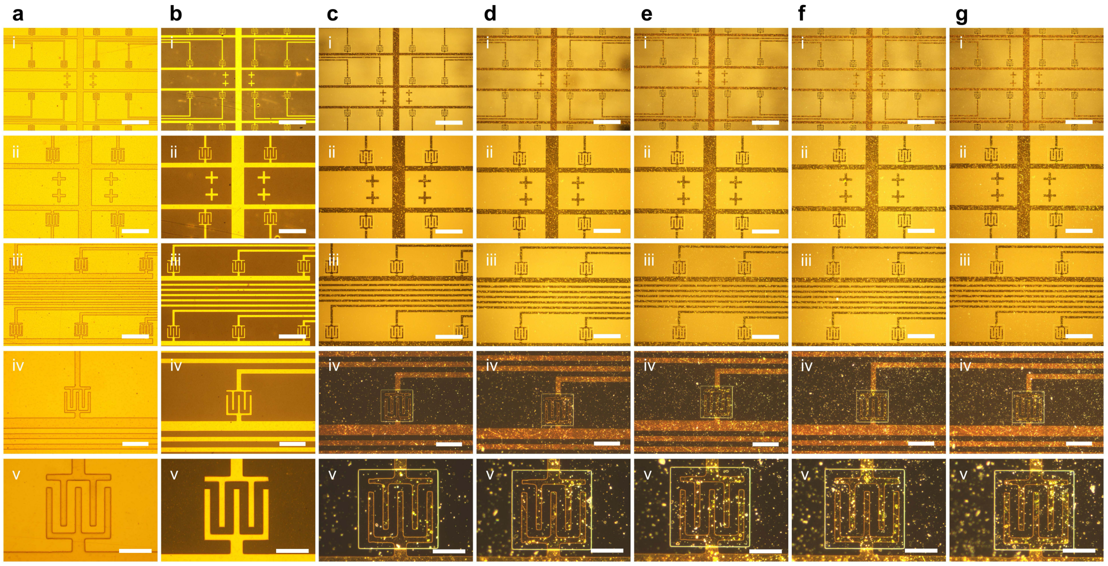

Supplementary Fig. 23 | Optical microscope images of the fabrication process of devices array. (**a**) Patterned photoresist after photolithography processing on thermal-evaporated Au, (**b**) Patterned Au electrode array after wet-etching, (**c**) S-CNO 2 mL film on Au array, (**d**) S-CNO 4 mL film on Au array, (**e**) S-CNO 6 mL film on Au array, (**f**) S-CNO 8 mL film on Au array, (**g**) S-CNO 10 mL film on Au array. Scale bar: (i) 400 μm, (ii) 200 μm, (iii) 200 μm, (iv) 100 μm, (v) 50 μm.

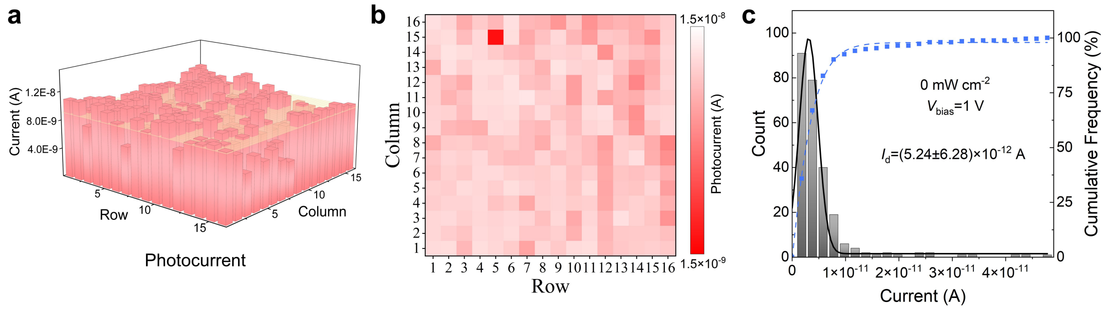

Supplementary Fig. 24 | Optoelectronic measurements of the device array. a, **b**, Spatial distribution (**a**) and photocurrents mapping (**b**) of photocurrents of the devices array under 1 V bias and 280 nm illumination. **c**, Statistical distribution of dark currents of the 256-pixel array in dark under 1 V bias. Note that, while exhibiting an average photocurrent of $(9.08 \pm 1.60) \times 10^{-9}$ A and an average dark current of $(5.24 \pm 6.28) \times 10^{-12}$ A, these flexible devices reveal an average responsivity of 0.29 A W^{-1} under 1 V bias and 280 nm illumination, which is very close to the that of rigid device (0.85 A W^{-1}) under the same test conditions.

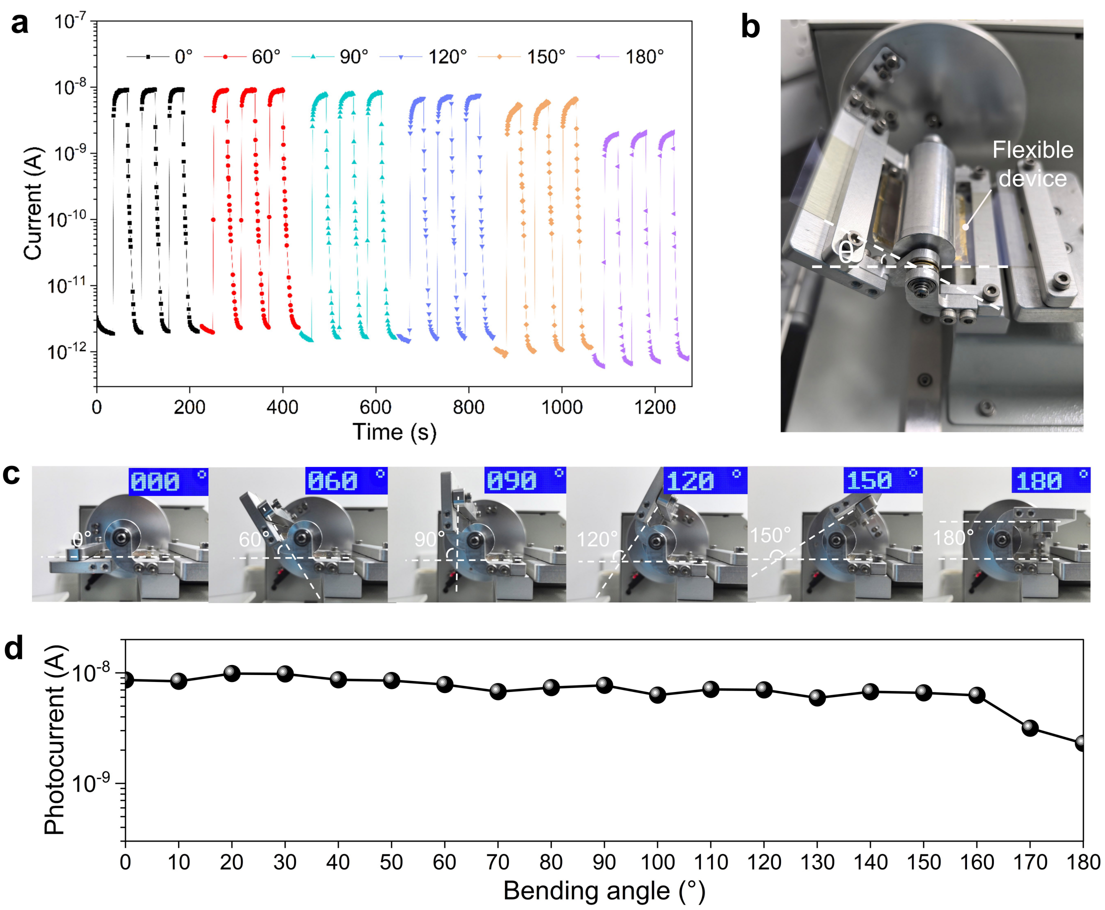

Supplementary Fig. 25 | Flexibility tests of the S-CNO film device. a, Semilogarithmic *I-t* curves measured at different bending angles under 1 V bias and 280 nm UV illumination. **b**, Optical image of the flexible device on a flexible electronic tester. **c**, Optical images of the bending test at different bending angles of 0°, 60°, 90°, 120°, 150° and 180°. **d**, Photocurrents of the device under bending angles from 0° to 180°. It can be seen that significant performance degradation of the device occurs when the bending angle is larger than 150°.

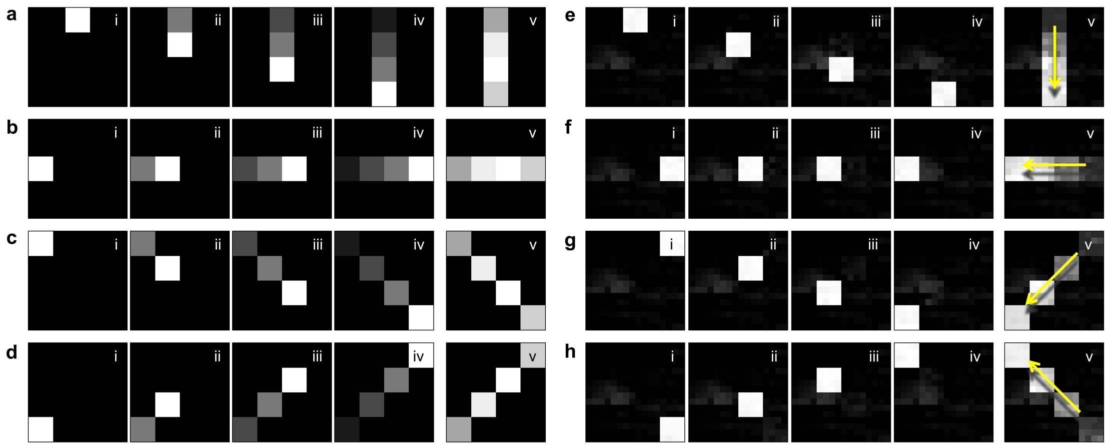

Supplementary Fig. 26 | Stimulated current maps resulted from D-CNO and S-CNO devices. Stimulated current maps in single-frame simulated by (**a**-**d**) single D-CNO device and (**e**-**h**) S-CNO devices array for motions in different directions. For single D-CNO device: (**a**) up to down, (**b**) left to right, (**c**) left-up to right-down, (**d**) left-down to right-up. For S-CNO devices array: (**e**) up to down, (**f**) right to left, (**g**) right-up to left-down, (**h**) right-down to left-up. Images: (i) single-frames at $t = t_1$, (ii) single-frames at $t = t_2$, (iii) single-frames at $t = t_3$, (iv) single-frames at $t = t_4$, (v) Time-weighted image with spatiotemporal information obtained from single-frames. For motions in eight directions, the processed images obtained from single-frames that produced by S-CNO device exhibit significant gray-level gradients, and the trained CNN model is able to recognize the motion trajectory with high accuracy, whereas the images obtained from the D-CNO device cannot be recognized.

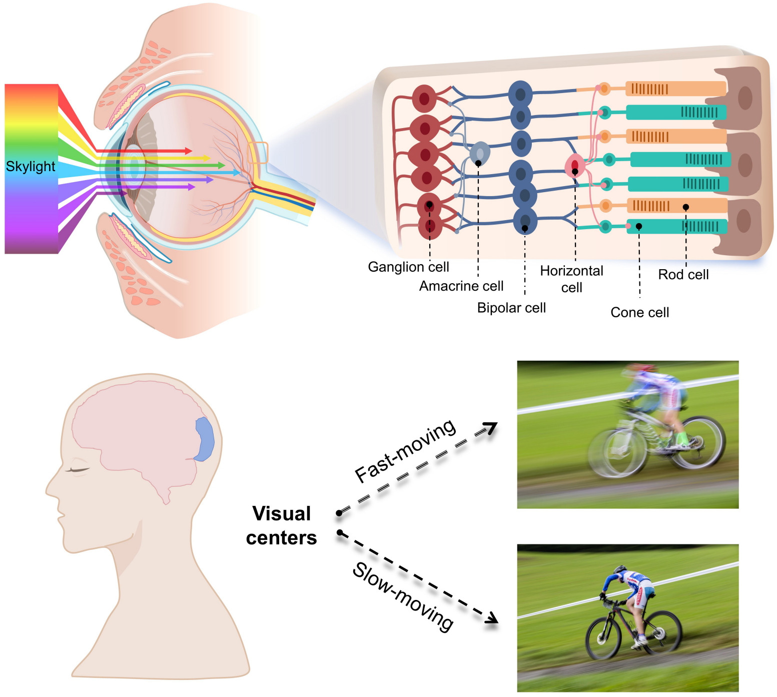

Supplementary Fig. 27 | Schematic illustration of the human visual system. Photoreceptor cells (rod and cone cells) in the retina convert light stimulus to graded action potential signals, which are subsequently transmitted to the visual cortex in the brain for further processing in conjunction with bipolar, ganglion, horizontal, and amacrine cells, generating coherent images. Visual persistence may occur due to the adaptation and recovery times (i.e., conformational changes after reversion after activation of the rhodopsin) of photoreceptor cells and associated neurons.

Supplementary Table 1. Representative two-dimensional semiconductors for photodetector in various wavebands

Material	Morphology	Synthesis method	Bandgap (eV)	Wave band	R (A W^{-1}) (wave -length)	D^* (Jones)	Large-area integration	Ref.
$Ca_2Nb_3O_{10}$	Nanosheets film	High-temperature calcination & Liquid exfoliation	3.61	UV	11.9 (280)	3.7×10^{14}	Y	This work
$Sr_2Nb_3O_{10}$	Single nanosheet	High-temperature calcination & Liquid exfoliation	3.90	UV	1214 (270)	1.4×10^{14}	N	4
BiOCl	Flake	Chemical vapor deposition	3.50	UV	35.7 (250)	2.2×10^{10}	N	5
$GeSe_2$	Monolayer	Chemical vapor transport reaction & Exfoliation	2.96	UV	0.2 (266)	1.6×10^{15}	N	6
GeSe	Layered crystal	Chemical vapor transport reaction & Exfoliation	1.60	SW-IR	0.005 (1310)	-	N	7
Graphene	Monolayer	Micromechanical exfoliation technique	-	Vis, NIR, MIR	8.61 (532)	-	N	8
MoS_2	Few-layer	Mechanical exfoliation	Tunable	Vis, NIR	2570 (635)	2.2×10^{12}	N	9
MoS_2	Monolayer	Chemical vapor deposition	-	Vis	3.4×10^{7} (520)	4.3×10^{16}	Y	10
MoS_2	Multilayer	Pulsed laser deposition	4.0	UV, Vis, NIR	1.96 (300)	-	Y	11
$MoSe_2$	Multilayer	Mechanical exfoliation	1.48	Vis, NIR	0.889 (520)	3.4×10^{14}	N	12
$MoTe_2$	Few-layer	Mechanical exfoliation	1.10	Vis, NIR	0.05 (637)	3.1×10^{9}	N	13
ReS_2	Multilayer	Mechanical exfoliation	1.53	Vis, IR	380 (800)	1.3×10^{10}	N	14
WS_2	Bilayer	Chemical vapor deposition	-	Vis	3.5 (532)	1.6×10^{10}	Y	15
WS_2	Monolayer	Chemical vapor deposition	~2.0	Vis	5.2×10^{-4} (532)	4.9×10^{9}	Y	16
WSe_2	Monolayer	Chemical vapor deposition	1.63	Vis	1.8×10^{5} (650)	10^{14}	Y	17
InSe	Few-layer	Mechanical exfoliation	1.30	Vis, NIR	12.3 (450)	1.1×10^{11}	N	18
In_2Se_3	Multilayer	Pulsed laser deposition	1.15	UV, vis, NIR	20.5 (532)	6.2×10^{11}	Y	19
Bi_2Te_3	Multilayer	Van der Waals epitaxy	0.3	IR	23.8 (1456)	8.0×10^{10}	Y	20

Supplementary Table 2. Summary of optoelectronic performances of the latest reported (within three years) and representative two-dimensional perovskites toward UV photodetection

Device structure	Morphology	Wavelength (nm)	On-off ratio	*R* (mA/W)	Rejection Ratio	D^* (Jones)	t_r / t_d	Flexibility	Large-area integration	Ref.
2D Perovskite oxide										
Au-$Ca_2Nb_3O_{10}$-Au	Nanosheets Film	280	1089595	11900	185560	3.71×10^{14}	42.4 μs / 1.77 ms	Y	Y	This work
Cr/Au-$Ca_2Nb_3O_{10}$-Cr/Au	Single Nanosheet	300	34000	14940	8840	8.7×10^{13}	80 μs / 5.6 ms	Y	N	21
Cr/Au-$Sr_2Nb_3O_{10}$-Cr/Au	Single Nanosheet	270	~10000	1214000	-	1.4×10^{14}	400 μs / 40 ms	Y	N	4
Cr/Au-$NdNb_2O_7$-Cr/Au	Single Nanosheet	260	1250	62000	9000	6.7×10^{12}	100 μs / 7.8 ms	N	N	22
Au-$Pb_2Nb_3O_{10}$-Ti	Single Nanosheet	350	700	2800	-	1.1×10^{12}	200 μs / 1.2 ms	N	N	23
Cr/Au-$NbWO_6$-Cr/Au	Single Nanosheet	290	155	378000	8840	-	1.05 ms / 88.3 ms	N	N	24
Cr/Au-KNb_3O_8-Cr/Au	Single Nanobelt	254	>1000000	30000	-	5.95×10^{11}	2.5 s / 1.8 s	N	N	25
Cr/Au-FM@$Ca_2Nb_3O_{10}$-Cr/Au	Film	300	200	80	3860	1.1×10^{12}	120 μs / 1.24 ms	Y	N	26
In-$Sr_2Nb_3O_{10}$-CuZnS-In	Film	300	>3000	0.28	2500	4.61×10^{10}	1.1 ms / 7.9 ms	N	N	27
Ag-NGQD-$Sr_2Nb_3O_{10}$-Ag	Film	270	22000	10.8	-	3.39×10^{11}	80 ms / 520 ms	N	N	2
Cr/Au-$Ca_2Nb_3O_{10}$-$Ti_3C_2T_x$ QD	Film	310	~100	264000	8516	-	-	Y	N	28
Cr/Au-$Ca_2Nb_{2.5}Ta_{0.5}O_{10}$-Cr/Au	Film	295	56000	469500	-	7.65×10^{13}	0.9 ms / 152 ms	N	N	29

Au-$Ca_2Nb_{3-x}Ta_xO_{10}$-$CsCu_2I_3$-Au	Film	250	~10000	81300	-	-	12.9 s / 1.82 s	Y	Y	30
FTO-$Ca_2Nb_{2.5}Ta_{0.5}O_{10}$-PC71BM-PH1000	Film	290	2200	60	-	~10^{13}	700 µs / 8.5 ms	N	Y	31
2D Metal halide perovskite										
Carbon-$(PA)_2PbBr_4$-Carbon	Single crystal	405	4150	2220	-	2.3×10^{13}	1.59 ms / 1.66 ms	N	N	32
Au-BA_2PbBr_4-Au	Single crystal	375	10000	1069	-	1.8×10^{13}	55 µs / 65 µs	N	N	33
Ag-BA_2PbBr_4-Ag	Single crystal	377	~10000	16.9	~1000	2.06×10^{12}	220 µs / 240 µs	N	N	34
Bi/Ag-PMA_2PbCl_4-Bi/Ag	Microbelt arrays	320	-	155.5	-	2.3×10^{6}	73 µs / 52 µs	Y	N	35
Bi/Ag-PMA_2PbCl_4-Bi/Ag	Microbelt	320	-	9000	-	1.01×10^{11}	162 µs / 226 µs	N	N	36
ITO-$(BA)_2MA_3Pb_4Br_{13}$-PC61BM-Bi/Ag	Film	360	-	1000	-	1.54×10^{14}	260 µs / 420 µs	N	N	37
Au-$(BBA)_2EA_2Pb_3Br_{10}$-Au	Single crystal	405	-	0.28	-	1.31×10^{10}	0.4 s / 4 s	N	N	38
Au-$(3AMPY)EAPb_2Br_7$-Au	Single crystal	405	23000	827.4	-	8.67×10^{12}	347 µs / 256 µs	N	N	39
Ag-$(BA)_2Pb(Cl_{0.1}Br_{0.9})_4$-Ag	Single crystal	340	-	3.19	-	3.87×10^{11}	232 ms / 252 ms	N	N	40
Au-$Cs_3Cu_2I_5$-Au	Nanosheet	365	-	2060	-	-	142 ms / 182 ms	N	N	41
		270	-	3780	-	-	163 ms / 203 ms			
Au-BP-BA_2PbBr_4-MoS_2-Au	Single crystal	405	-	17.7	-	1.5×10^{11}	38 µs / 50 µs	N	N	42
Cr/Au-SnP_2S_6-PEA_2PbI_4-Cr/Au	Single crystal	365	~10000	67100	-	2.8×10^{11}	30 µs / 120 µs	N	N	43

Supplementary Note I. Film-formation processes in D-CNO and S-CNO films

(I) D-CNO film fabricated by traditional strategy: In previous studies, after the droplet is simply drop-coated onto the substrate, the nanosheets settle and stack in a disordered and haphazard way with the volatilization of the solvent, eventually forming a defect-rich film with a rough and uneven surface (Supplementary Fig. 3).

(II) S-CNO film fabricated by the COAF process: For processes i-vi in Fig. 1a: (i) The large number of tiny micro-droplets ejected from the gun ensure a uniform distribution of nanosheets, and as they cover the surface of the substrate, they also ensure the large-are in-plane spreading parallel to the substrate. (ii) For each individual micro-droplet, as the top-down exfoliation endows CNO nanosheets with intrinsic negative charges, the resultant repulsive forces prevent the aggregation of nanosheets, further contributing to their dispersion inside the droplet. (iii) Upon the droplets contact the substrate, there will be repulsive forces between the nanosheets in the droplets and those on the substrate. The closer (farther) the distance, the stronger (weaker) the repulsive forces. The non-uniform repulsive forces cause the nanosheets in the droplets to rotate and end up approximately parallel to the nanosheets deposited on the substrate to balance the repulsive forces. (iv) Since the prepared nanosheets precursor solution contains nanosheets with lateral size ranging from a few micrometers (lager ones) to tens of nanometers (tinier ones), it is also necessary to consider the behavior and significant role of tinier nanosheets in the COAF process – as the solvent evaporates and penetrates, they will fill in the gaps and voids to ensure the integrality of the films (details shown in Supplementary Fig. 5). Note that there are two main forces of the void-filing process: a. the same repulsion between the nanosheets (preventing tinier nanosheets from settling directly on the surface of larger nanosheets), b. the percolation effect of the solvent accompanied by volatilization. Meanwhile, the larger nanosheets gradually settle in this ordered arrangement as the solvent dissipates. However, considering the irregular shape of the nanosheets, there may still be some inevitable voids inside the nanosheets films. (v) After the solvent completely evaporates, the layer-by-layer stackings of nanosheets in each individual micro-droplet ensure high degrees of orientation and tight structures. At the same time, due to the large-area and uniform

coverage of the substrate surface by a large number of micro-droplets during the spray-coating process, the nanosheets inside the droplets will connect with the adjacent ones to form a block of thin film while settling, forming the large-area in-plane spreading of nanosheets parallel to the substrate surface. (vi) Ultimately, the ordered stack and the connections covering the entire substrate of the nanosheets simultaneously completes the construction of the highly oriented film.

Supplementary Note II. Thickness-related photodetection performance

The light absorption of 2D semiconductors is essential for the performance of photodetectors (such as photoresponsivity and speed), as well as film-based devices. Photoresponsivity R_λ of a photoconductor can be expressed by[44,45]

$$R = R_0 \cdot G = \frac{\eta_q e}{h\upsilon} \cdot G \qquad (1)$$

where R_0 is the intrinsic responsivity, G is the photoconductive gain, η_q is the quantum efficiency, e is the electron charge, h is the Planck constant, and υ is the light frequency. The quantum efficiency η_q is proportional to the absorbed light power P_a, which can be expressed by

$$P_a = P_0(1 - e^{-\alpha d}) \qquad (2)$$

where P_0 is the incident light power, α is the absorption coefficient, and d is the thickness of the film. Therefore, the thin thickness of the film (e.g. S-CNO 4 mL film) is unfavorable for the absorption of incident light. Due to the increase in film-thickness d caused by the spraying process, more light is absorbed and the generation of more carriers is stimulated, causing the photocurrent and responsivity to gradually increase with thickness. However, as d continues to increase, photoinduced electrons and holes in films need to travel longer distances to be collected by electrodes. Combined with incremental defects in films, many carriers may recombine before they reach the electrodes, ultimately resulting in the decrease in photocurrent and responsivity. In summary, photocurrent and responsivity increase and then decrease with increasing S-CNO film thickness (Supplementary Fig. 17).

Supplementary Note III. Performance characterization of the CNO photodetector

(I) Photosensitivity: The device-photosensitivity-evaluation parameters on-off ratio, responsivity (R_λ) and detectivity (D^*) are calculated from

$$on-off\ ratio=\frac{I_{\mathrm{photo}}}{I_{\mathrm{dark}}} \tag{3}$$

$$R_\lambda=\frac{I_{\mathrm{photo}}-I_{\mathrm{dark}}}{P_\lambda\cdot S} \tag{4}$$

$$D^*=\frac{R_\lambda}{\sqrt{2eI_{\mathrm{dark}}/S}} \tag{5}$$

where I_{photo} and I_{dark} represent the photocurrent and dark current, P_λ and S denote the light power density and effective illustration area and e is the electron charge. The photocurrents of the device were measured under monochromatic light illumination with wavelengths varying from 230 to 550 nm at 5 V bias. Meanwhile, as the photogeneration probability of carriers is proportional to the absorbed photon flux, the photocurrent is delineated as a power function of the light power density, which is expressed as

$$I=\alpha P^{\theta} \tag{6}$$

where α is a wavelength-dependent constant and θ is a parameter related to the drift, transmit, trapping, and recombination process of carriers (Supplementary Fig. 17e).

(II) Photoresponse speed: Rise time (t_{r}) and decay time (t_{d}) estimated from pulse response characterization are utilized to evaluate the photoresponse speed of a photodetector, which is mainly related to the transmission and collection process of charge carriers. t_{r} and t_{d} are defined as the time taken by 10% to 90% of the net photocurrent.

$$|t_{\mathrm{r}},t_{\mathrm{d}}|=|t_{90\%}-t_{10\%}| \tag{7}$$

The response time in a photoconductive device is related to carrier mobility, channel length, and applied bias[46]. Note that the decay time (t_{d}) is fitted by the biexponential decay curve expressed as

$$I=A_1\cdot e^{-t/\tau_1}+A_2\cdot e^{-t/\tau_2}+I_0 \tag{8}$$

where A_1 and A_2 are the related constants and τ_1 and τ_2 are the time constants (Supplementary Fig. 18). In this work, the transient response time was measured under the excitation of a 10 Hz pulse laser (355 nm) at 1 V bias.

(III) SCLC test: As shown in Supplementary Fig. 19, the ohmic and trap-filled limit regions are distinctively separated at trap-filled limit voltage (V_{TFL}). The trap density (n_{trap}) can then be determined by the V_{TFL} with[47-49]

$$n_{\text{trap}} = \frac{2\varepsilon_0\varepsilon_r V_{\text{TFL}}}{eL^2} \quad (9)$$

where ε_0 denotes the vacuum permittivity of 8.85×10^{-12} F m^{-1}, ε_r is the dielectric constant of $Ca_2Nb_3O_{10}$ (ε_r =26)[50], *e* represents the elementary charge of 1.6×10^{-19} C, and *L* is the film thickness.

(IV) Statistical analysis: Coefficient of variation (C_V) that reflects the discrete degree of the statistical data (e.g. photocurrent and dark current) is calculated by

$$C_{\text{V}} = \frac{|\sigma|}{\mu} = \frac{\sqrt{\frac{1}{N}\sum_{i=1}^{N}(X_{\text{i}}-\mu)}}{\frac{1}{N}\sum_{i=1}^{N}X_{\text{i}}} \quad (10)$$

where μ and σ denote the mean value and standard deviation of collected data.

Supplementary Note IV. Shallow-trap dominance-induced performance balance

Experiments have shown that when being illuminated, the change in carriers/resistance (corresponding to the decay time) within a photoconductive semiconductor consists of three main procedures with different timescales: (i) recombination of carriers occurring in less than approximately 10^{-3} s, (ii) de-trapping process happened in shallow traps within about 10^{-3} ~10 s, (iii) de-trapping process happened in deep traps within about 10 ~ 10^4 s (Supplementary Fig. 20)[51,52].

A trap is amphoteric as it can act both as an electron trap and as a hole trap. The actual state of occupation of the trap determines which of the above it function as. Assuming that the trap is monovalent, for an empty trap which is ready to receive an electron, it acts as an electron trap. And for an electron-containing trap that is ready to receive a hole, it is a hole trap[53]. Typically, carriers trapped in trap states remain in the state until certain conditions are met (e.g. thermal or photo-excitation) before they are released. During this time, the carriers may slow down or stop moving and no longer participate in the conductive process. It is hard for electrons to return to the ground state, thus prolonging the response time. As multiple trapping occurs in both shallow traps and deep traps, the decay time of a photoconductor can be expressed by[51,52,54]

$$\tau_{\mathrm{decay}} = \tau_{\mathrm{r}} + \tau_{\mathrm{t}}(1+\rho) \tag{11}$$

in which

$$\frac{1}{\tau_{\mathrm{t}}} = S_{\mathrm{p}} N_{\mathrm{V}} v exp\left(\frac{E_{\mathrm{V}}-E_{\mathrm{trap}}}{kT}\right) \tag{12}$$

where τ_r is the recombination time (life time of carriers), τ_t is the de-trap time (i.e. the time for emptying traps from taps), ρ is the probability that a carrier gets re-trapped before recombination, S_p is the cross section for capture of carriers in traps, N_v is the effective density of states in the valence bands, v is the arithmetic mean thermal velocity, E_v and E_{trap} represent the energy level of the trap state and the valence band, k is Boltzmann's constant, and T is the absolute temperature. Therefore, a small change in E_{trap} leads to a change in the de-trap time by orders of magnitude, and the trapping and de-trapping processes of deep trap states with large energy level differences will greatly increase the reponse time. Generally, extended trap state lifetime lowers the

photoresponse speed of the device, and it can further lead to undesired long lag in images when applied in imaging applications. Therefore, it is of great significance to modulate the response speed of the CNO nanosheet thin-film device.

In a photoconductor, the localized states introduced by defect states, impurity energy levels, and interfacial states have significant effects on processes such as carrier trapping, de-trapping and recombination[54]. Under illumination, the quasi-Fermi levels move toward their respective band edges[44], and these localized states between E_c and E_v may function as recombination centers or trap states depending on their position (Supplementary Fig. 21). Subsequently, states above the electron quasi-Fermi level and below the hole quasi-Fermi level usually serve as electron and hole trap states, respectively, while states in between will turn into recombination centers[53]. The electron quasi-Fermi level and the hole quasi-Fermi level are expressed as

$$E_{F_n} = E_F + kT \ln\left(\frac{n}{n_0}\right) \quad E_{F_n} = E_F + kT \ln\left(\frac{n}{n_0}\right) \tag{13}$$

where E_F is the Fermi level, n_0 and p_0 are the equilibrium densities of the electron and hole, and n and p are the densities of the electron and hole under illumination, respectively. For an n-type semiconductor such as CNO under illumination, the locations of the localized states, recombination centers and trap states are shown in Supplementary Fig. 21.

Liquid-exfoliated CNO nanosheets contain a large number of defects and surface traps. The extra energy levels in the energy band structure of the D-CNO film are usually associated with the presence of defects and traps that can trap and release carriers. The slow rise in the *I-t* curve of the D-CNO device when exposed to light corresponds to the trapping process of carriers, and the subsequent platform indicates that the trap states are basically filled up. But in S-CNO films, the decremental concentration of defects and traps means that there are fewer additional energy levels, thus, electrons are more likely to occupy higher energy states, resulting in an increase in the quasi-Fermi energy level of electrons. Correspondingly, the hole quasi-Fermi energy level decreases, as shown in Fig 3f. In the D-CNO film which contains a large number of defects and traps, the quasi-Fermi level for holes is located near the deep

trap states, in which carriers take much longer to be trapped or released. While in S-CNO film, the layer-by-layer oriented assembly of nanosheets leads to a slump of traps, especially decremental interfacial traps. As electrons are more likely to occupy higher energy states, the quasi-Fermi energy level of electron increases. Correspondingly, the hole quasi-Fermi energy level decreases. Therefore, many original deep traps are transformed into recombination centers. Due to the predominance of shallow traps and the increase in the number of recombination centers, the overall trapping and de-trapping time of carriers is greatly reduced, resulting in a significantly faster photoresponse speed in S-CNO devices.

Supplementary Note V. Visual persistence phenomenon - Palinopsia

The visual system is one of the most important perceptual organs of a person, as more than 80% of the environmental information is provided by visual perception[55]. When we observe a fast-moving target, it may be perceived that the object becomes blurred and produces ghosting, the so-called visual persistence phenomenon, or pathological palinopsia (Supplementary Fig. 27)[56]. Palinopsia, which means the persistence of visual perceptions after the excitatory stimulus is removed, describes a various of visual symptoms that cannot be easily classified as either visual illusions or visual hallucinations. Palinopsia contains symptoms such as visual trailing (discrete or blurred images caused by moving object, also called ghosting), physiological afterimages (afterimages lacking clarity appear after accepting a bright stimulus and moving visual focus), prolonged indistinct afterimage (indistinct or unformed afterimage in the location in the visual field as the original stimulus), and light streaking (motion between a person and a light source causes persisting streaks for several seconds)[56]. In human vision systems, visual persistence is commonly caused by dysfunction in light perception or motion perception, such as dysregulated cortico-thalamo-retinal feedback (former dysfunction)[57] and visual trailing (latter dysfunction)[58].

This phenomenon is characterized by the persistence of visual images after the excitatory stimulus disappears, which originates from the adaptation and recovery process of photoreceptor cells in the retina. Similarly, such time-critical issues in artificial vision systems have attracted tremendous attention in image contrast enhancement, noise reduction, event-driven data acquisition, and other visual data preprocessing methods for future simulation and substitution of biological vision systems[59-61]. The image captured by an artificial sensor is occasionally indistinct due to delayed response time and the relative motion between the sensor and the target object, which seriously affects the subsequent image processing and recognition accuracy. Some conventional electronic image stabilization techniques solve the problem between frames using image processing algorithms, but they require additional resources for computation and storage[62]. Instead, improving the performance of hardware, i.e., photodetectors in the light-sensing module of a vision system, is

conducive to directly overcoming the ghosting issue and obtaining high-quality single-frame images for accurate visual information processing[63].

Supplementary References

1. Mo, X. et al. Highly-efficient all-inorganic lead-free 1D $CsCu_2I_3$ single crystal for white-light emitting diodes and UV photodetection. *Nano Energy* **81**, 105570 (2021).
2. Liu, X. et al. Enhanced response speed in 2D perovskite oxides-based photodetectors for UV imaging through surface/interface carrier-transport modulation. *ACS Appl. Mater. Interfaces* **14**, 48936-48947 (2022).
3. Wen, Z. et al. Graphene induced structure and doping level tuning of evaporated $CsPbBr_3$ on different substrates. *Chem. Eng. J.* **452**, 139243 (2023).
4. Li, S., Zhang, Y., Yang, W., Liu, H. & Fang, X. 2D perovskite $Sr_2Nb_3O_{10}$ for high-performance UV photodetectors. *Adv. Mater.* **32**, 1905443 (2020).
5. Han, W. et al. Atomically thin oxyhalide solar-blind photodetectors. *Small* **16**, 2000228 (2020).
6. Yan, Y. et al. Direct wide bandgap 2D $GeSe_2$ monolayer toward anisotropic UV photodetection. *Adv. Opt. Mater.* **7**, 1900622 (2019).
7. He, T. et al. Extrinsic photoconduction induced short-wavelength infrared photodetectors based on Ge-based chalcogenides. *Small* **17**, 2006765 (2021).
8. Zhang, B. Y. et al. Broadband high photoresponse from pure monolayer graphene photodetector. *Nat. Commun.* **4**, 1811 (2013).
9. Wang, X. et al. Ultrasensitive and broadband MoS_2 photodetector driven by ferroelectrics. *Adv. Mater.* **27**, 6575-6581 (2015).
10. Seo, J. et al. Ultrasensitive photodetection in MoS_2 avalanche phototransistors. *Adv. Sci.* **8**, 2102437 (2021).
11. Jiao, L. et al. Layer-dependent photoresponse of 2D MoS_2 films prepared by pulsed laser deposition. *J. Mater. Chem. C* **7**, 2522-2529 (2019).
12. Huang, Y. et al. High-performance broadband visible-near infrared photodetector

enabled by atomic capping layer. *Adv. Opt. Mater.* **10**, 2200539 (2022).
13. Huang, H. et al. Highly sensitive visible to infrared $MoTe_2$ photodetectors enhanced by the photogating effect. *Nanotechnology* **27**, 445201 (2016).
14. Xiang, D. et al. Anomalous broadband spectrum photodetection in 2D rhenium disulfide transistor. *Adv. Opt. Mater.* **7**, 1901115 (2019).
15. Tan, H. et al. Ultrathin 2D photodetectors utilizing chemical vapor deposition grown WS_2 with graphene electrodes. *ACS Nano* **10**, 7866-7873 (2016).
16. Lan, C. et al. Wafer-scale synthesis of monolayer WS_2 for high-performance flexible photodetectors by enhanced chemical vapor deposition. *Nano Res.* **11**, 3371-3384 (2018).
17. Zhang, W. et al. Role of metal contacts in high-performance phototransistors based on WSe_2 monolayers. *ACS Nano* **8**, 8653-8661 (2014).
18. Tamalampudi, S. R. et al. High performance and bendable few-layered InSe photodetectors with broad spectral response. *Nano Lett.* **14**, 2800-2806 (2014).
19. Zheng, Z. Q., Yao, J. D. & Yang, G. W. Growth of centimeter-scale high-quality In_2Se_3 films for transparent, flexible and high performance photodetectors. *J. Mater. Chem. C* **4**, 8094-8103 (2016).
20. Wang, F. et al. Submillimeter 2D Bi_2Se_3 flakes toward high-performance infrared photodetection at optical communication wavelength. *Adv. Funct. Mater.* **28**, 1802707 (2018).
21. Zhang, Y. et al. High-performance two-dimensional perovskite $Ca_2Nb_3O_{10}$ UV photodetectors. *Nano Lett.* **21**, 382-388 (2021).
22. Zhang, Y. et al. Two-dimensional perovskite $NdNb_2O_7$ for high-performance UV photodetectors by a general exfoliation and assembly strategy. *Nano Energy* **117**, 108915 (2023).
23. Zhang, Y. et al. Two-dimensional perovskite $Pb_2Nb_3O_{10}$ photodetectors. *J. Mater. Sci. Technol.* **164**, 95-101 (2023).
24. Song, P., Zhang, Y., Wang, J., Liu, H. & Tian, L. Novel two-dimensional $NbWO_6$ nanosheets for high performance UV photodetectors. *Adv. Electron. Mater.* (2023).
25. Ping, Y. et al. Polarization sensitive solar-blind ultraviolet photodetectors based

on ultrawide bandgap KNb_3O_8 nanobelt with fringe-like atomic lattice. *Adv. Funct. Mater.* **32**, 2111673 (2022).

26. Zhang, Y. et al. Integration of filter membrane and $Ca_2Nb_3O_{10}$ nanosheets for high performance flexible UV photodetectors. *J. Mater. Sci. Technol.* **129**, 108-114 (2022).
27. Zhang, J. & Liu, J. Two-dimensional perovskite $Sr_2Nb_3O_{10}$ nanosheets meet CuZnS film: Facile fabrications and applications for high-performance self-powered UV photodetectors. *J. Alloys Compd.* **908**, 164594 (2022).
28. Zheng, Y. et al. MXene quantum dots/perovskite heterostructure enabling highly specific ultraviolet detection for skin prevention. *Matter* **6**, 506-520 (2023).
29. Liu, X. et al. Boosted responsivity and tunable spectral response in B-site substituted 2D $Ca_2Nb_{3-x}Ta_xO_{10}$ perovskite photodetectors. *Adv. Funct. Mater.* **31**, 2101480 (2021).
30. Chen, J. et al. Work-function-tunable MXenes electrodes to optimize p-$CsCu_2I_3$/n-$Ca_2Nb_{3-x}Ta_xO_{10}$ junction photodetectors for image sensing and logic electronics. *Adv. Funct. Mater.* **32**, 2201066 (2022).
31. Yan, T. et al. Large-area 2D perovskite oxides/organic heterojunction enables highly-sensitive self-powered photodetector for ultraviolet light communication. *Adv. Funct. Mater.* **34**, 2311042 (2023).
32. Dong, K. et al. 2D perovskite single-crystalline photodetector with large linear dynamic range for UV weak-light imaging. *Adv. Funct. Mater.* **34**, 2306941 (2023).
33. Gao, L., Luo, X., Sun, J. L., Li, Q. & Yan, Q. Room-temperature solvent evaporation induced crystallization: A general strategy for growth of halide perovskite single crystals by applying the Le Chatelier's principle. *Small* **19**, 2303687 (2023).
34. Liang, L. et al. Ultraviolet photodetection with low detection limit using inch-sized two-dimensional hybrid perovskite crystal. *Adv. Opt. Mater.* **10**, 2201342 (2022).
35. Guo, L. et al. Patterned 2D ferroelectric perovskite single-crystal arrays for self-

powered UV photodetector boosted by combining ferro-pyro-phototronic and piezo-phototronic effects. *Nano Lett.* **22**, 8241-8249 (2022).

36. Guo, L. et al. Ferro-pyro-phototronic effect in monocrystalline 2D ferroelectric perovskite for high-sensitive, self-powered, and stable Ultraviolet Photodetector. *ACS Nano* **16**, 1280-1290 (2022).

37. Guo, L. et al. 2D Ruddlesden-Popper perovskite ferroelectric film for high-performance, self-powered and ultra-stable UV photodetector boosted by ferro-pyro-phototronic effect and surface passivation. *Nano Energy* **102**, 107714 (2022).

38. Tang, L. et al. Photoexcited ultraviolet-to-infrared (II) pyroelectricity in a 2D ferroelectric perovskite driving broadband self-powered photoactivities. *Adv. Funct. Mater.* **33**, 2214858 (2023).

39. Fu, D. et al. Bilayered Dion-Jacobson hybrid perovskite bulk single crystals constructed with aromatic diammonium for ultraviolet-visible-near-infrared photodetection. *Chem. Mater.* **35**, 2541-2548 (2023).

40. Wang, Q. et al. Two-dimensional hybrid perovskite crystals for highly sensitive and stable UV light detector. *Opt. Mater.* **145**, 114408 (2023).

41. Lv, J. et al. Epitaxial growth of lead-free 2D $Cs_3Cu_2I_5$ perovskites for high-performance UV photodetectors. *Small* **18**, 2201715 (2022).

42. Qiao, B. S. et al. Photosensitive dielectric 2D perovskite based photodetector for dual wavelength demultiplexing. *Adv. Mater.* **35**, 2300632 (2023).

43. Zhang, Y. et al. 2D Ruddlesden-Popper perovskite sensitized SnP_2S_6 ultraviolet photodetector enabling high responsivity and fast speed. *Nanoscale Horiz.* **8**, 108-117 (2022).

44. Konstantatos, G. & Sargent, E. H. PbS colloidal quantum dot photoconductive photodetectors: transport, traps, and gain. *Appl. Phys. Lett.* **91**, 173505 (2007).

45. Liu, E. et al. High responsivity phototransistors based on few-layer ReS_2 for weak signal detection. *Adv. Funct. Mater.* **26**, 1938-1944 (2016).

46. Qiu, Q. & Huang, Z. Photodetectors of 2D materials from ultraviolet to terahertz waves. *Adv. Mater.* **33**, 2008126 (2021).

47. Dong, S. et al. All-inorganic perovskite single-crystal photoelectric anisotropy.

Adv. Mater. **34**, 2204342 (2022).

48. Chen, J. et al. Single-crystal thin films of cesium lead bromide perovskite epitaxially grown on metal oxide perovskite ($SrTiO_3$). *J. Am. Chem. Soc.* **139**, 13525-13532 (2017).
49. Liu, Y. et al. A 1300 mm^2 ultrahigh-performance digital imaging assembly using high-quality perovskite single crystals. *Adv. Mater.* **30**, 1707314 (2018).
50. Zhang, P. et al. Flexible high-performance microcapacitors enabled by all-printed two-dimensional nanosheets. *Sci. Bull.* **67**, 2541-2549 (2022).
51. Jiang, J. et al. Defect engineering for modulating the trap states in 2D photoconductors. *Adv. Mater.* **30**, 1804332 (2018).
52. Hornbeck, J. A. & Haynes, J. R. Trapping of minority carriers in silicon. 1. p-type silicon. *Physical Review* **97**, 311-321 (1955).
53. Simmons, J. G. & Taylor, G. W. Nonequilibrium steady-state statistics and associated effects for insulators and semiconductors containing an arbitrary distribution of traps. *Phys. Rev. B* **4**, 502-511 (1971).
54. Streetman, B. G. Carrier recombination and trapping effects in transient photoconductive decay measurements. *J. Appl. Phys.* **37**, 3137-3144 (1966).
55. Farrow, K. et al. Ambient illumination toggles a neuronal circuit switch in the retina and visual perception at cone threshold. *Neuron* **78**, 325-338 (2013).
56. Gersztenkorn, D. & Lee, A. G. Palinopsia revamped: a systematic review of the literature. *Surv. Ophthalmol.* **60**, 1-35 (2015).
57. McLelland, D., Baker, P. M., Ahmed, B. & Bair, W. Neuronal responses during and after the presentation of static visual stimuli in macaque primary visual cortex. *J. Neurosci.* **30**, 12619-12631 (2010).
58. Kilpatrick, Z. P. & Bard Ermentrout, G. Hallucinogen persisting perception disorder in neuronal networks with adaptation. *J. Comput. Neurosci.* **32**, 25-53 (2012).
59. Mennel, L. et al. Ultrafast machine vision with 2D material neural network image sensors. *Nature* **579**, 62-66 (2020).
60. Chai, Y. In-sensor computing for machine vision. *Nature* **579**, 32-33 (2020).

61. Seung, H. et al. Integration of synaptic phototransistors and quantum dot light-emitting diodes for visualization and recognition of UV patterns. *Sci. Adv.* **8**, eabq3101 (2022).

62. Lotter, W., Kreiman, G. & Cox, D. A neural network trained for prediction mimics diverse features of biological neurons and perception. *Nat. Mach. Intell.* **2**, 210-219 (2020).

63. Cho, S. W., Jo, C., Kim, Y.-H. & Park, S. K. Progress of materials and devices for neuromorphic vision sensors. *Nano-micro Lett.* **14**, 203 (2022).
